# Complete mitogenome of *Pelecorhynchus vulpes* (Macquart) (Diptera: Pelecorhynchidae) and its phylogenetics implications on Tabanomorpha

**DOI:** 10.7717/peerj.21470

**Published:** 2026-08-24

**Authors:** Christian R. González, Mónica Saldarriaga-Córdoba

**Affiliations:** 1 Instituto de Entomologia, Facultad de Ciencias Básicas, Universidad Metropolitana de Ciencias de la Educación, Santiago, Región Metropolitana, Chile; 2 Programa de Doctorado en Salud de Ecosistemas y Sostenibilidad & Centro de Investigación en Recursos Naturales y Sustentabilidad, Universidad Bernardo O’Higgins, Santiago, Chile

**Keywords:** Mitochondrial genome, Phylogeny, Phylogenetic analyses, Tabanomorpha

## Abstract

The infraorder Tabanomorpha represents a key lineage within the lower Brachycera; however, the phylogenetic placement of several families, including Pelecorhynchidae, remains incompletely resolved owing to the lack of mitogenomic data. In this study, we sequenced and characterized the complete mitochondrial genome of *Pelecorhynchus vulpes* (Macquart, 1850), representing the first mitogenome reported for both the genus and the family. The circular genome is 15,390 bp in length and comprises a typical set of 37 genes, including 13 protein-coding genes, 22 transfer RNA genes, two ribosomal RNA genes, and a control region. The genome exhibits a strong A+T bias (78.4%) and retains the ancestral insect gene arrangement. Comparative analyses revealed a high degree of structural conservation with other Tabanomorpha, particularly in Tabanidae and Athericidae. Phylogenetic analyses based on concatenated mitochondrial protein-coding and ribosomal genes, using both Bayesian inference and maximum likelihood approaches, support Pelecorhynchidae as a sister lineage to Athericidae and Tabanidae. These findings provide new genomic evidence supporting the phylogenetic placement of Pelecorhynchidae and contribute to understanding of the evolutionary relationships within Tabanomorpha. Additionally, the phylogenetic affinity between the Chilean and Australasian representatives of *Pelecorhynchus* is consistent with a Gondwanan origin for the group, although alternative scenarios, such as long-distance dispersal, cannot be excluded. This study highlights the importance of mitogenomic data from underrepresented lineages and provides information for new integrative studies that combine mitochondrial and nuclear data to resolve the evolutionary history of Tabanomorpha.

## Introduction

Tabanomorpha includes the families Tabanidae, Rhagionidae, Athericidae, Pelecorhynchidae, Bolbomyiidae, Vermilionidae, Austroleptidae, and Oreoleptidae (Wiegmann & Yeates, 2017). This infraorder includes approximately 5,900 species and represents a diverse lineage of large flies that plays a key role in the evolutionary history of Diptera (Wiegmann & Yeates, 2017). The monophyly of Tabanomorpha is supported by both morphological (Yeates, 2002) and molecular evidence (Song, Yi & Yin, 2022). Morphological characteristics include the presence of a brush on the larval mandible, a retractile larval head, a convex and bulbous adult clypeus, and a ventrolaterally expanded first cercal segment in adults (Wiegmann et al., 2000). Molecular analyses have corroborated this monophyly (Wiegmann et al., 2003; Wiegmann et al., 2011). However, the clade (Stratiomyomorpha + (Xylophagomorpha + Tabanomorpha)) typically exhibits low to moderate support (Shin et al., 2018), and recently, Yuan & Chen (2025) proposed a new topology for brachyceran infraorders: (Tabanomorpha + (Xylophagomorpha + (Stratiomyomorpha + Muscomorpha))). The taxonomic position and limits of the family Pelecorhynchidae have been controversial. Enderlein (1922) and Enderlein (1925) considered the species now placed in *Pelecorhynchus* as a subfamily within Tabanidae, assigning them to two genera: *Coenura* Bigot, 1857 and *Pelecorhynchus*. Ferguson (1926) followed Enderlein’s proposal (1922, 1925) and included *Pelecorhynchus* in the subfamily Pelecorhynchinae (Tabanidae). Later, Mackerras & Fuller (1942) elevated this group to the family rank. Steyskal (1953) considered *Pelecorhynchus* to be closely related to *Coenomyia* Latreille and placed these genera in Coenomyiidae (now included in Xylophagidae). Teskey (1970) related *Glutops* Burgess to *Pelecorhynchus* based on morphological similarities between larvae and pupae. Nagatomi (1982) maintained that Pelecorhynchidae comprises only *Pelecorhynchus*. Subsequently, Woodley (1989) and Sinclair (1992) placed *Pelecorhynchus*, *Pseudoerinna* Shiraki, and *Glutops* in Pelecorhynchidae based on synapomorphic features in both adults and larvae. Stuckenberg (2001) recognized all three genera as members of the subfamily Pelecorhynchinae within Rhagionidae. Molecular evidence support Teskey’s proposition that *Glutops* and *Pelecorhynchus* form a monophyletic group (Wiegmann et al., 2000; Wiegmann et al., 2003). Kerr (2010) showed that Pelecorhynchidae is sister to Athericidae and Tabanidae. Finally, Morita et al. (2016) recovered Pelecorhynchidae together with Oreoleptidae as sister to Athericidae and Tabanidae.

Pelecorhynchidae comprises 54 species in four genera: *Glutops* Burgess, *Pseudoerinna* Shiraki, *Pelecorhynchus* Macquart, and *Coenura* Bigot (González et al., 2023). These genera are morphologically diverse (Krivosheina, 1997) and are distributed in the Nearctic, Palearctic, Australian, and Neotropical regions (González & Elgueta, 2020). The genus *Pelecorhynchus* is known only in the Australasian (Australia and Tasmania) (Daniels, 1989) and Andean (central and southern Chile) (González & Elgueta, 2020) regions. *Pelecorhynchus vulpes* (Macquart) is a rare species found in south-central Chile, characterized by reddish-brown coloration. The scutum is reddish-brown with abundant orange-rufous setulae and without longitudinal stripes. The scutellum is reddish-brown with abundant setulae of the same color on the distal margin. The pleura has abundant orange-rufous setulae, and the anepisternum has tufts of long whitish setulae. The wings are slightly infuscated orange. The abdomen has orange rufous setulae without spots. In a recent study on Chilean species distribution, González et al. (2023) reported that *P. vulpes* and two other species from Chile formed a clade with the Australian species *P. personatus* (Walker) based on both morphological and molecular results. Future studies, particularly those including all Australian species, could provide the information needed to test whether *P. vulpes* should be kept under *Pelecorhynchus* or placed in a different taxon.

Although previous molecular analyses have clarified the relationships within Tabanomorpha, the phylogenetic placement of Pelecorhynchidae remains uncertain, largely because of the limited availability of molecular data and the absence of complete mitogenomes for the group. Complete mitogenome sequences have become a tool for resolving higher-level phylogenetic relationships in Diptera, as they provide extensive and relatively conserved molecular characters compared to those of individual gene fragments (Cameron, 2014; Song, Yi & Yin, 2022). Moreover, the inclusion of mitogenome data from Pelecorhynchidae could offer new insights into both the evolutionary systematics and Gondwanan biogeographic history of this lineage, given its disjunct distribution across Australasia and southern South America. In this study, we sequenced and characterized the complete mitogenome of *Pelecorhynchus vulpes* to clarify the phylogenetic position of Pelecorhynchidae within the Tabanomorpha.

## Material and Methods

### Sample collection, morphological identification

The *P. vulpes* specimen was collected from Isla Teja (−39,805503 S/ −73,264320 W), Región de Los Ríos, Chile. The voucher specimen was identified follows the key of Llanos, González & Saldarriaga (2015). The specimen has been deposited in the Instituto de Entomología, Universidad Metropolitana de Ciencias de la Educación (IEUMCE).

### DNA extraction, sequencing and assembly

Genomic DNA was extracted from the muscle tissue of the legs of the individual identified above using the Qiagen^®^ DNeasy Blood & Tissue commercial kit, following the manufacturer’s instructions, and stored at −4 °C for subsequent sequencing in Macrogen SA.

Genomic sequencing was performed using an Illumina NovaSeq 6000 PE150 platform. Whole Genome Shotgun (WGS) libraries with a 350 bp insert size were prepared. Raw reads were filtered using the CUTADAPT version 2.10 software (Martin, 2011) to remove adapter sequences and sequences below the Q30 quality threshold. Reads shorter than 70 bases and singletons were excluded. A *de novo* assembly was performed with all clean reads in the NOVOPlasty v4.3.3 assembler (Dierckxsens, Mardulyn & Smits, 2017) using the seed input *cox1* sequence from *Atylotus miser* (NC_030000). Within the assembled scaffolds, the mitogenome was identified using the BLASTN tool by comparison with a custom database of selected mitochondrial genomes from organisms belonging to the order Diptera.

### Mitogenome annotation and characteristics analysis

Preliminary annotation of the sequence was performed using MitoZ v3.6 (Meng et al., 2019) with an invertebrate mitochondrial genetic code (translation table 5). Manual curation was subsequently performed to correct the reading frames and validate gene predictions through BLAST comparisons with closely related species. The tRNA Scan-SE 2.0 server (Chan & Lowe, 2019) and RNAfold web server (Lorenz et al., 2011) were used to re-identify the tRNA and to reconfirm their secondary structure. In cases in which these tools failed to detect certain tRNAs, their genomic locations were inferred by aligning the corresponding regions with homologous sequences from phylogenetically related taxa. The mitogenome map was illustrated using Geneious Prime version 2025.2.1, which was created by Biomatters (https://www.geneious.com). Tandem repeat elements in the A + T-rich region were identified using Tandem Repeats Finder (http://tandem.bu.edu/trf/trf.html) (Benson, 1999).

### Phylogenetic analysis

A total of 29 species were used in the phylogenetic analysis, including two outgroup species from Asilidae and Stratiomyidae. The species used in this study are listed in Table S1. In this study, we used the partial or complete mitochondrial genomes of 27 species of Tabanomorpha (26 retrieved from GenBank and one generated in this study) along with *Leptogaster longicauda* (Asilidae) and *Ptecticus aurifer* (Stratiomyidae) as outgroups (Table 1) for phylogenetic analysis. PhyloSuite v 1.2.3 (Zhang et al., 2020) was used for data standardization and information extraction. The 13 protein-coding genes (PCGs) were aligned using the MACSE algorithm (Ranwez et al., 2011) with the invertebrate mitochondrial genetic code. The two rRNAs were aligned using MAFFT with the G-INS-I strategy. Gaps and ambiguous sites were removed from the alignments using the Gblocks (Castresana, 2000; Talavera & Castresana, 2007) algorithm in PhyloSuite 1.2.3 under the default settings. Alignments of each individual gene were concatenated using PhyloSuite 1.2.3. The maximum likelihood (ML) tree was reconstructed using IQ-TREE v.1.6.8 (Nguyen et al., 2015) and the substitution models were estimated using ModelFinder (Kalyaanamoorthy et al., 2017) implemented in the IQ-TREE package with the Bayesian information criterion (BIC). Branch support was estimated using 10,000 replicates of the ultrafast bootstrap. Bayesian inference (BI) trees were reconstructed using MrBayes 3.2.7 (Ronquist & Huelsenbeck, 2003) and fitting substitution models were determined in MEGA v 11.0.13 (Tamura, Stecher & Kumar, 2021) with the BIC criterion. BI analysis using the default settings by four simultaneous Markov chains was run for five million generations in two independent runs, with sampling every 1,000 generations, and the initial 25% of samples were discarded as burn-in.

**Table 1:** *P. vulpes* mitochondrial genome. Organization of the *P. vulpes* mitochondrial genome. Note: in intergenic nucleotide “–” indicate overlapping between genes.

**Name**	**Start**	**Stop**	**Size (bp)**	**Strand**	**Anticodon**	**Start Codon**	**Stop Codon**	**Intergenic nucleotides**
**trnI**	388	452	65	Forward (+)	GAU			−3
**trnQ**	450	518	69	Reverse (-)	UUG			0
**trnM**	519	586	68	Forward (+)	CAU			0
***nad2***	587	1,616	1030	Forward (+)		ATG	T	0
**trnW**	1,617	1,684	68	Forward (+)	UCA			−9
**trnC**	1,676	1,741	66	Reverse (-)	GCA			−1
**trnY**	1,741	1,805	65	Reverse (-)	GUA			1
***cox1***	1,807	3,337	1,531	Forward (+)		CGA	T	0
**trnL2**	3,338	3,402	65	Forward (+)	UAA			3
***cox2***	3,406	4,092	687	Forward (+)		ATG	TAA	1
**trnK**	4,094	4,164	71	Forward (+)	CUU			−1
**trnD**	4,164	4,227	64	Forward (+)	GUC			0
***atp8***	4,228	4,386	159	Forward (+)		ATT	TAA	−7
***atp6***	4,380	5,055	676	Forward (+)		ATG	T	0
***cox3***	5,056	5,843	788	Forward (+)		ATG	TA	−1
**trnG**	5,843	5,908	66	Forward (+)	UCC			0
***nad3***	5,909	6,262	354	Forward (+)		ATT	TAA	3
**trnA**	6,266	6,329	64	Forward (+)	UGC			−1
**trnR**	6,329	6,392	64	Forward (+)	UCG			0
**trnN**	6,393	6,462	70	Forward (+)	GUU			1
**trnS1**	6,464	6,528	65	Forward (+)	GCU			1
**trnE**	6,530	6,595	66	Forward (+)	UUC			19
**trnF**	6,615	6,677	63	Reverse (-)	GAA			0
***nad5***	6,678	8,394	1,717	Reverse (-)		ATT	T	13
**trnH**	8,410	8,473	64	Reverse (-)	GUG			1
***nad4***	8,475	9,813	1,339	Reverse (-)		ATG	T	−7
***nad4l***	9,807	10,103	297	Reverse (-)		ATG	TAA	2
**trnT**	10,106	10,168	63	Forward (+)	UGU			0
**trnP**	10,169	10,234	66	Reverse (-)	UGG			2
***nad6***	10,237	10,759	523	Forward (+)		ATT	T	1
***cytb***	10,761	11,895	1,135	Forward (+)		ATG	T	0
**trnS2**	11,896	11,962	67	Reverse (+)	UCA			6
***nad1***	11,969	12,917	949	Reverse (-)		ATT	T	10
**trnL1**	12,928	12,992	65	Reverse (-)	UAG			0
**rrnL**	12,993	14,315	1,323	Reverse (-)				0
**trnV**	14,316	14,387	72	Reverse (-)	UAC			0
**rrnS**	14,388	15,169	788	Reverse (-)				2
**OH**	15,172	293	∼510					

## Results and Discussion

### Mitogenome organization and base composition

**Figure 1:**
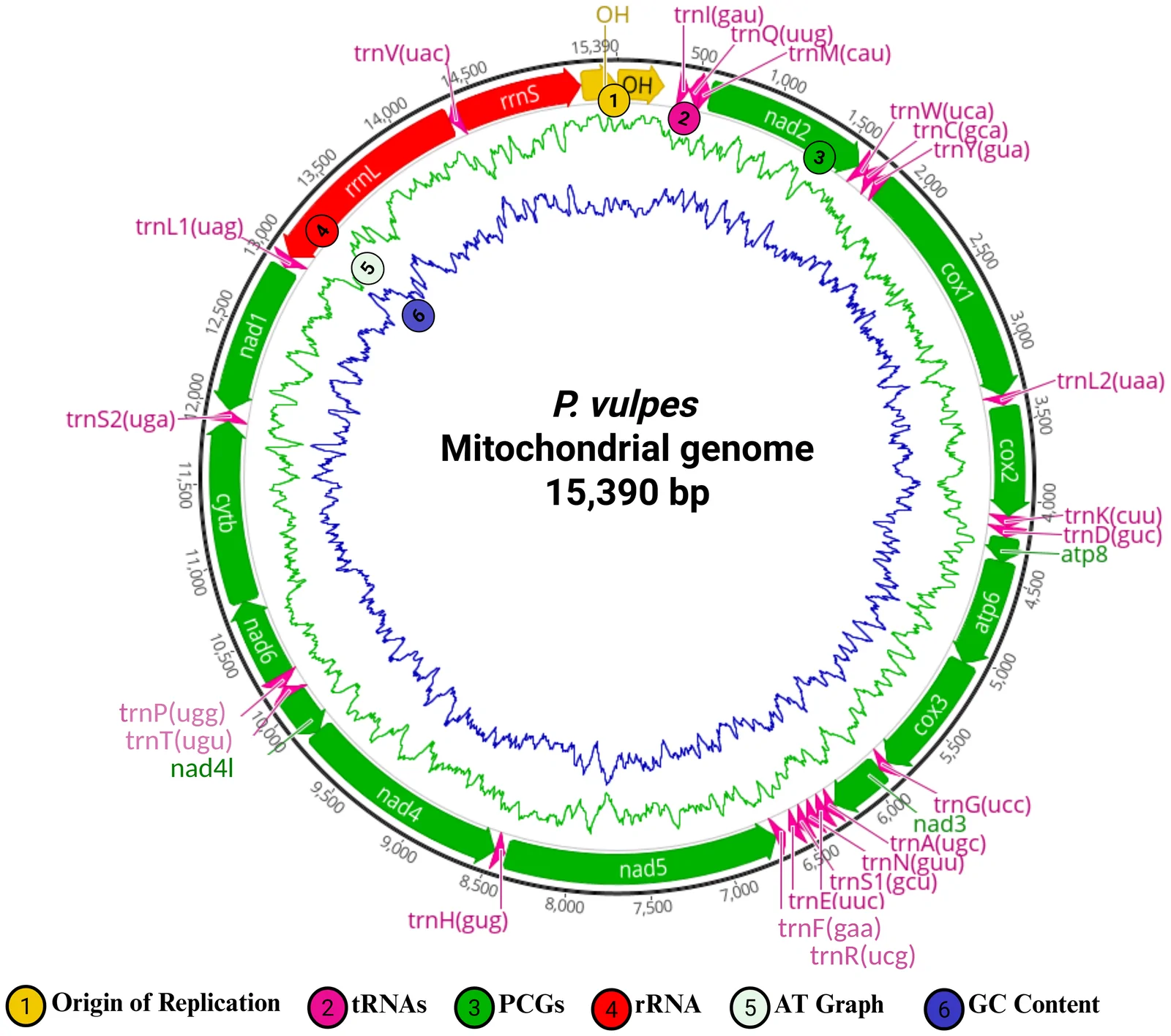
Circular representation of the mitogenome of *Pelecorhynchus vulpes* depicting the gene order. PCGs, ribosomal RNA genes are shown with standard abbreviations. Gene orientations are indicated by arrow directions.

The sequence generated in this study was deposited in GenBank under accession number PV955036. The mitogenome sequence of *Pelecorhynchus vulpes* is 15,390 bp in length (Fig. 1) and consists of 37 typical genes, compressed by two rRNA (small subunit ribosomal RNA and large subunit ribosomal RNA), 22 tRNA genes, 13 PCGs, and a partial sequence of the control region (510 bp) (Fig. 1 and Table 1). The size of the control region (also known as the D-loop) in insect mitogenomes varies significantly between species. Typically, it ranges from 883 base pairs (bp) to 1,424 bp in some insects (Wang et al., 2016; Fu et al., 2021; Li & Li, 2022; Liu et al., 2022). Our results indicate that *the P. vulpes* mitogenome shows the same synteny as that proposed for the mitogenomes of Insecta (Cameron, 2014).

The heavy chain (positive strand) of *P. vulpes* encoded 23 genes (nine PCGs and 14 tRNAs), while the light chain (negative strand) encoded 14 genes (eight tRNAs, two rRNA, four PCGs). A detailed analysis of the gene boundaries in the *P. vulpes* mitochondrial genome revealed three distinct junction patterns: eight genes exhibited overlapped with adjacent genes (ranging from one to nine bp), fifteen genes were separated by intergenic spacers (1–19 bp), and fourteen genes were immediately adjacent, showing neither overlap nor spacing. In these cases, one gene ended and the next began at consecutive nucleotide positions (Table 1). This heterogeneous organization reflects the compact and variable architecture of the insect mitochondrial genomes (Song, Wei & Wang, 2023; Xing, Kang & Zhao, 2023). Eight overlapping regions were identified, with the longest overlap (nine bp) located between *trnW* and *trnC*. A conserved overlapping sequence of eight bp (AAGCCTTA) is frequently found between these genes in orthorrhaphan flies (Wang et al., 2016); however, in *P. vulpes*, the overlapping region extends to nine bp and presents a single base substitution (TAAGCTTTA), reflecting both conservation and variation within this motif. Among the 15 intergenic spacers (totalling 61 bp), the longest (19 bp) was found between *trnE* and *trnF*, closely matching the ∼20 bp spacing commonly observed in other dipterans (Xing, Kang & Zhao, 2023). The second-longest intergenic region (13 bp) was located between *nad5* and *trnH*. Notably, the genome also contains a characteristic 7 bp overlap between *atp8* and *atp6*, a feature widely conserved across insect mitochondrial genomes, including those of other Brachycera species (Wang et al., 2016; Fu et al., 2021). Additionally, a conserved seven bp overlap (TTAACAT) between *nad4* and *nad4L* was detected, which is consistent with the pattern observed in many insects (Xing, Kang & Zhao, 2023). These findings contrast with patterns in other species, such as *Atylotus miser* (Tabanidae), where gene overlaps range from 1 to 8 bp and intergenic regions tend to be shorter. Wang et al. (2016) also reported exceptions to the conserved *trnW–trnC* overlap in *Rhamphomyia insignis* and *Suragina* sp., where 24 bp and 8 bp intergenic regions, respectively, were observed in place of overlaps.

Similar to other insects, the mitochondrial genome of *P. vulpes* exhibits a strong nucleotide bias toward adenine and thymine (Wang et al., 2024; Medina et al., 2025). This compositional asymmetry is typically assessed using the AT content, AT skew, and GC skew. In *P. vulpes*, the AT content reached 78.4% (A: 41.1%, T: 37.3%), whereas the GC content was 21.6% (G: 8.5%, C: 13.1%). The calculated AT skew was 0.0483, indicating a slight excess of adenine over thymine, and the GC skew was −0.2135, reflecting a higher proportion of cytosine than guanine.

**Figure 2:**
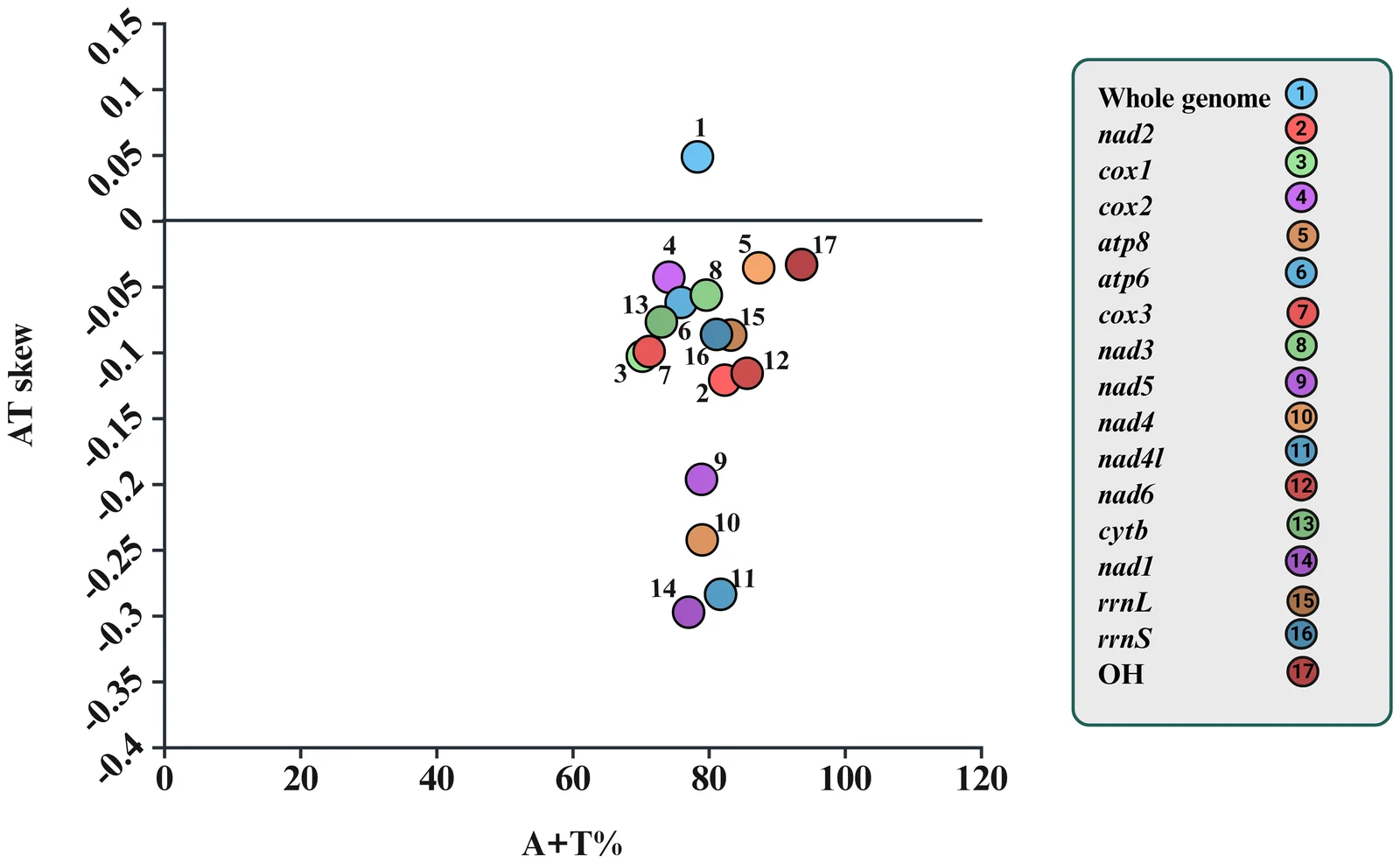
Information on AT content and AT skew in the whole mitogenome, PCGs, and rRNAs of *P. vulpes*.

The A+T content of all protein-coding and rRNA genes in the mitogenome was higher than the G+C content. In most genes and in the control region, the A+T content was also higher than that in the complete mitogenome, except for *cox1*, *cox2*, *atp6*, *cox3*, *cytb*, and *nad1* (Table 2 and Fig. 2). This result is consistent with the known nucleotide composition bias characteristic of insect mitogenomes (Liu et al., 2022; Xing, Kang & Zhao, 2023).

**Table 2:** Nucleotide composition and AT/GC skew values of PCGs and rRNA genes in the mitochondrial genome of *P. vulpes*. Note: Positive AT skew indicates A > T; negative, T > A. Positive GC skew indicates G > C; negative, C > G.

**Region**	**A%**	**C%**	**G%**	**T%**	**A+T%**	**G+C%**	**AT skew**	**GC skew**
Whole genome	41.1	13.1	8.5	37.3	78.4	21.6	0.0483	−0.2135
*nad2*	36.2	11.1	6.5	46.2	82.4	17.6	−0.1213	−0.2597
*cox1*	31.5	15.3	14.3	38.8	70.3	29.7	−0.1031	−0.0352
*cox2*	35.5	15.0	10.8	38.7	74.2	25.8	−0.0431	−0.1638
*atp8*	42.1	10.1	2.5	45.3	87.4	12.6	−0.0360	−0.6000
*atp6*	35.7	15.1	8.9	40.4	76.0	24.0	−0.0623	−0.2593
*cox3*	32.1	16.4	12.3	39.2	71.3	28.7	−0.0996	−0.1416
*nad3*	37.6	12.4	7.9	42.1	79.7	20.3	−0.0567	−0.2222
*nad5*	31.7	7.5	13.5	47.3	79.0	21.0	−0.1967	0.2833
*nad4*	29.9	6.9	14.0	49.1	79.1	20.9	−0.2427	0.3429
*nad4l*	29.3	5.4	12.8	52.5	81.8	18.2	−0.2840	0.4074
*nad6*	37.9	9.2	5.2	47.8	85.7	14.3	−0.1161	−0.2800
*cytb*	33.7	15.2	11.6	39.4	73.1	26.9	−0.0771	−0.1344
*nad1*	27.5	8.7	14.2	49.5	77.1	22.9	−0.2976	0.2636
*rrnL*	38.0	5.4	11.3	45.3	83.3	16.7	−0.0871	0.3575
*rrnS*	37.1	6.4	12.4	44.1	81.2	18.8	−0.0866	0.3197
OH	45.3	3.5	2.8	48.4	93.7	6.3	−0.0335	−0.1111

We analyzed the AT and GC skew at different levels (in the complete mitochondrial genome, across protein-coding genes (PCGs) and tRNAs). A positive AT skew was observed only at the whole mitogenome level (0.0483), indicating a global bias toward adenine over thymine. In contrast, the GC skew was negative (−0.2135), reflecting a higher proportion of cytosine relative to guanine. This compositional bias has also been reported in other dipterans such as *Delia antiqua* (Zhang et al., 2015). At the level of individual PCGs, all genes exhibited a negative AT skew, while GC skew values varied among genes. Notably, a positive GC skew was observed in *nad5*, *nad4*, *nad4l*, *nad1*, and both RNA genes, indicating a relative enrichment of guanine over cytosine in these regions (Table 2, Fig. 2). A similar pattern was reported by Medina et al. (2025) in tephritid flies.

### Protein coding genes and codon usage

In the mitochondrial genome of *P. vulpes*, 13 protein-coding genes (PCGs) span a total of 11,185 bp and are arranged in the typical insect gene order. Most PCGs use the conventional start codons ATT or ATG. One exception is *cox1*, which initiates with the non-canonical start codon CGA, a feature that has also been reported in other dipterans by Xing, Kang & Zhao (2023) and in Lepidoptera by Liu et al. (2016). Regarding termination, most genes end with the canonical stop codon TAA. Some PCGs encode a complete TAA stop codon, whereas others are presumed to be completed post-transcriptionally *via* polyadenylation (Ojala, Montoya & Attardi, 1981).

**Figure 3:**
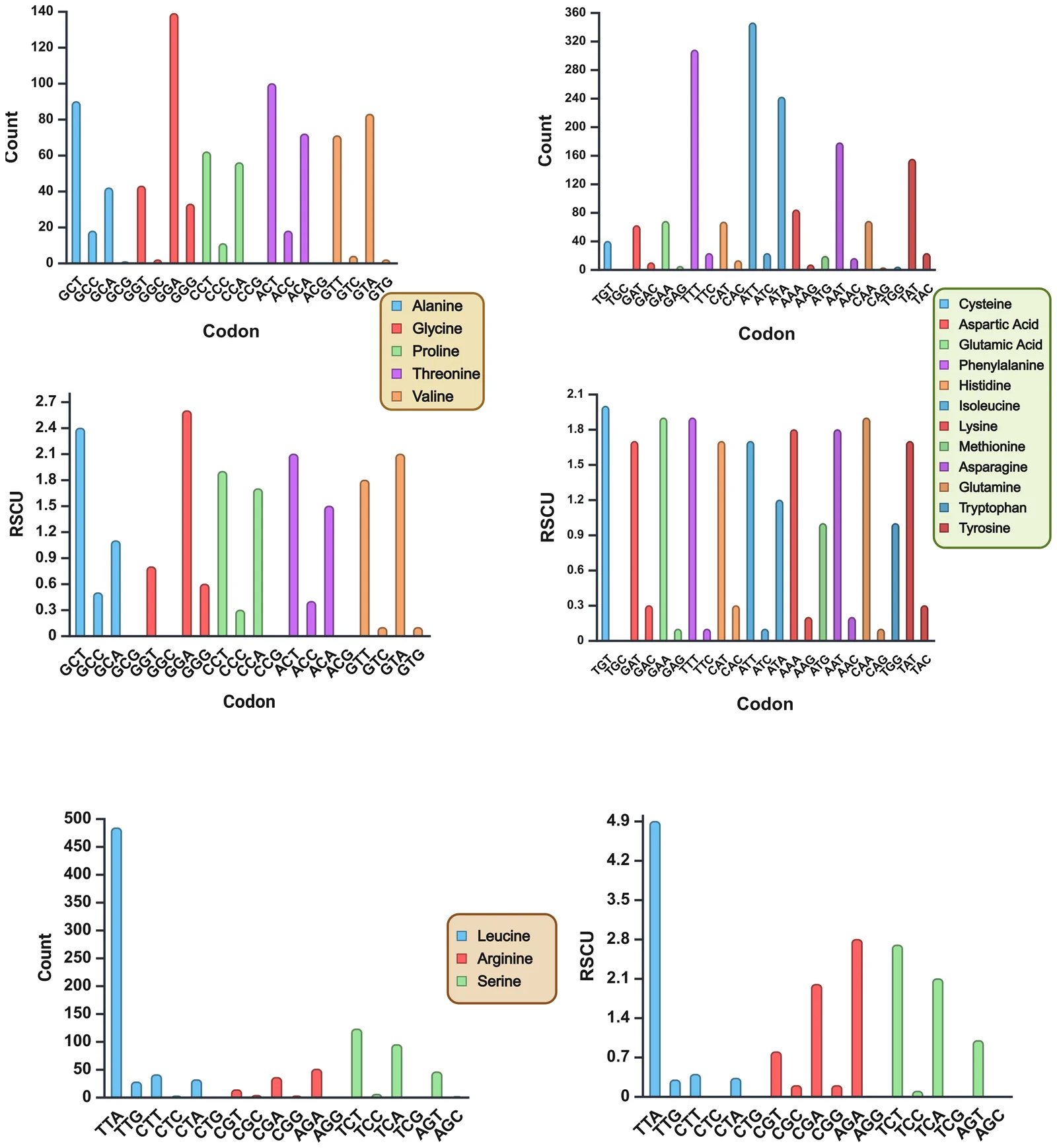
Codon counts and relative synonymous codon usage (RSCU) of the 13 protein-coding genes (PCGs) in the mitochondrial genome of *Pelecorhynchus vulpes* collected in Valdivia, Chile. Codons are grouped according to their corresponding amino acid, indicated by the colored boxes. Each color represents a different amino acid.

Nine PCGs (*nad2, cox1, cox2, atp8, atp6, cox3, nad3, nad6*, and *cytb*) were encoded on the positive strand and four PCGs (*nad5, nad4, nad4l, and nad1*) were encoded on the negative strand. The A + T bias is also reflected in the relative codon usage of the PCGs (Wang et al., 2016). The codon number and relative synonymous codon usage (RSCU) of the PCGs are summarized in Fig. 3, showing that the three amino acids with the highest frequency (number) were phenylalanine, isoleucine, and leucine, and the most prevalent codons were A + T-rich codons, such as UUU (Phe), AUU (Ile), and UUA (Leu), which were comprised of U and A.

### Ribosomal and transfer RNA genes

Nucleotide composition analysis of the ribosomal RNA genes (*rrnL* and *rrnS*) in the mitochondrial genome of *P. vulpes* revealed a high A+T content, consistent with the overall AT-richness characteristic of insect mitogenomes. Both genes showed negative AT skew values, indicating relative enrichment of thymine over adenine. In contrast, GC skew values were positive for both rRNAs, reflecting a higher frequency of guanine compared to cytosine (Table 2). This compositional asymmetry suggests a conserved strand bias in mitochondrial rRNAs, which is likely associated with structural and functional constraints within the mitochondrial ribosome.

Like other insects, *Pelecorhynchus vulpes* contains 22 tRNAs genes, which range from 63 bp (trnF and trnT) to 72 bp (trnV) (Table 1), with a total length of 1,456 bp. Fourteen tRNA were situated in the positive strand and nine were positioned on the negative strand. Analysis of AT and GC skew in the mitochondrial tRNA genes of *P. vulpes* revealed a heterogeneous pattern. Unlike the complete mitogenome, where the AT skew was consistently positive, individual tRNAs exhibited positive, negative, and near-neutral AT skew values, indicating no conserved bias toward adenine or thymine across these genes. A similar variability was observed for the GC skew, with values ranging from positive to negative, suggesting differential enrichment of guanine or cytosine depending on the specific tRNA (Table 3). These results highlight the lack of strand-specific compositional bias conservation in the tRNA set, in contrast to the trends typically observed in coding regions. This contrasts with the findings of Medina et al. (2025) in tephritid flies, where tRNA genes showed more consistent patterns, with a positive AT skew toward adenine (0.0274 ± 0.0148) and a negative GC skew toward cytosine (−0.1092 ± 0.0283).

**Table 3:** Mitochondrial tRNAs in *P. vulpes*. Nucleotide composition and AT/GC skew values of mitochondrial tRNAs in *P. vulpes*. Note: Positive AT skew indicates A > T; negative, T > A. Positive GC skew indicates G > C; negative, C > G.

**tRNA**	**A%**	**C%**	**G%**	**T%**	**A+T%**	**G+C%**	**AT skew**	**GC skew**
trnI(gau)	36.9	9.2	15.4	38.5	75.4	24.6	−0.0204	0.2500
trnQ(uug)	36.2	5.8	14.5	43.5	79.7	20.3	−0.0909	0.4286
trnM(cau)	33.8	16.2	11.8	38.2	72.1	27.9	−0.0612	−0.1579
trnW(uca)	45.6	11.8	10.3	32.4	77.9	22.1	0.1698	−0.0667
trnC(gca)	42.4	7.6	13.6	36.4	78.8	21.2	0.0769	0.2857
trnY(gua)	35.4	9.2	16.9	38.5	73.8	26.2	−0.0417	0.2941
trnL2(uaa)	36.9	9.2	15.4	38.5	75.4	24.6	−0.0204	0.2500
trnK(cuu)	38.0	15.5	14.1	32.4	70.4	29.6	0.08	−0.0476
trnD(guc)	51.6	4.7	4.7	39.1	90.6	9.4	0.0	0.2833
trnG(ucc)	48.5	7.6	9.1	34.8	83.3	16.7	0.1636	0.0909
trnA(ugc)	29.7	9.4	17.2	43.8	73.4	26.6	−0.1915	0.2941
trnR(ucg)	31.3	18.8	14.1	35.9	67.2	32.8	−0.0698	−0.1429
trnN(guu)	45.7	8.6	12.9	32.9	78.6	21.4	0.1636	0.2000
trnS1(gcu)	38.5	10.8	10.8	40.0	78.5	21.5	−0.0196	0.0000
trnE(uuc)	40.9	7.6	4.5	47.0	87.9	12.1	−0.0690	−0.2500
trnF(gaa)	42.9	4.8	15.9	36.5	79.4	20.6	0.08	0.5385
trnH(gug)	39.1	4.7	12.5	43.8	82.8	17.2	−0.0566	0.4545
trnT(ugu)	41.3	9.5	11.1	38.1	79.4	20.6	0.04	0.0769
trnP(ugg)	40.9	4.5	10.6	43.9	84.8	15.2	−0.0357	0.4
trnS2(uga)	44.8	7.5	13.4	34.3	79.1	20.9	0.1321	0.2857
trnL1(uag)	38.5	4.6	12.3	44.6	83.1	16.9	−0.0741	0.4545
trnV(uac)	40.3	8.3	11.1	40.3	80.6	19.4	0.0000	0.1429

**Figure 4:**
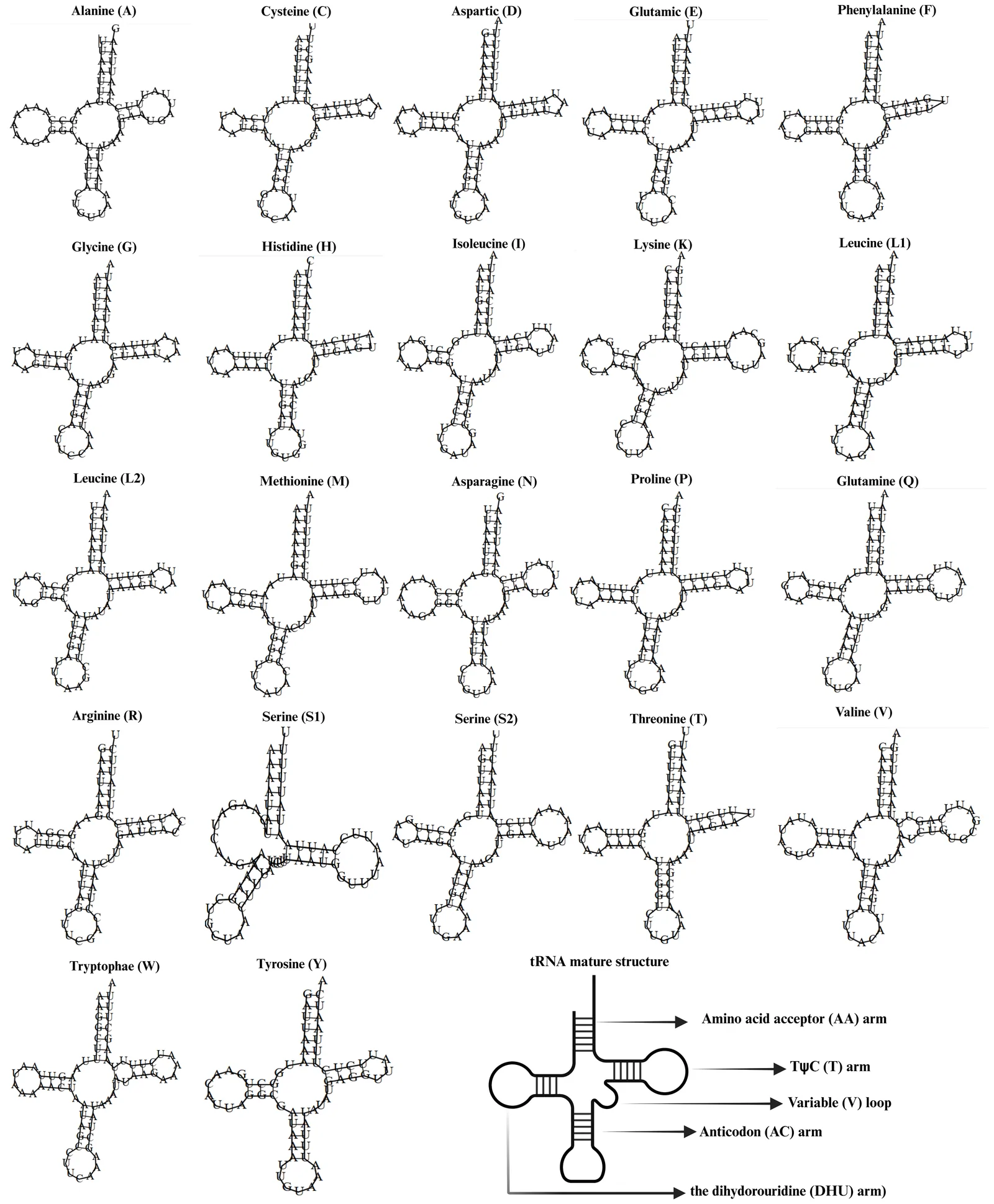
Predicted secondary cloverleaf structure of the tRNAs of the *P. vulpes* mitogenome. The tRNAs are labelled with the abbreviations of their corresponding amino acids.

Most tRNAs genes could be folded into the typical clover-leaf secondary structure (Fig. 4), whereas *trnS1* lacks a DHU stem and loop; the latter has been observed for this gene in other metazoan mitochondrial genomes (Wolstenholme, 1992). In spider Pons et al. (2019) identified 18 tRNAs that showed the loss of one arm, either the TΨC (C, D, E, F, G, H, I, K, L1, L2, N, M, P, T, V, and W) or the DHU (Q and S2).

### Phylogenetic inferences

Phylogenetic analyses were conducted using a concatenated dataset comprising the 13 mitochondrial PCGs and the two ribosomal RNA genes. After removing alignment gaps and ambiguous sites, the final dataset contained 11,069 nucleotide positions. The mitogenome sequences of twenty-seven Tabanomorpha species were included, representing the families Athericidae (*Suragina* sp.), Pelecorhynchidae (*Pelecorhynchus vulpes*), Rhagionidae (*Chrysopilus cristatus, Desmomyia sinensis, Rhagio lineola*, *Rhagio* sp. and *Symphoromyia crassicornis*), Tabanidae (*Atylotus miser*, *A. sinensis, Chrysops dissectus, C. niger* and *C. silvifascies*, and *Cydistomyia duplonotata*, *Haematopota subcylindrical*, *H. pluvialis*, *H. tukestanica*, and *H. vexatina*, *Hybomitra bimaculate*, *Tabanus amaenus, T. autumnalis, T. chrysurus, T. formosiensis,* and *T. haysi*), and Xylophagidae (*Coenomyia ferruginea*, *Dialysis* sp., and *Heterostomus curvipalpis*). *Leptogaster longicauda* (Asilidae) and *Ptecticus aurifer* (Stratiomyidae) were used as outgroups.

**Figure 5:**
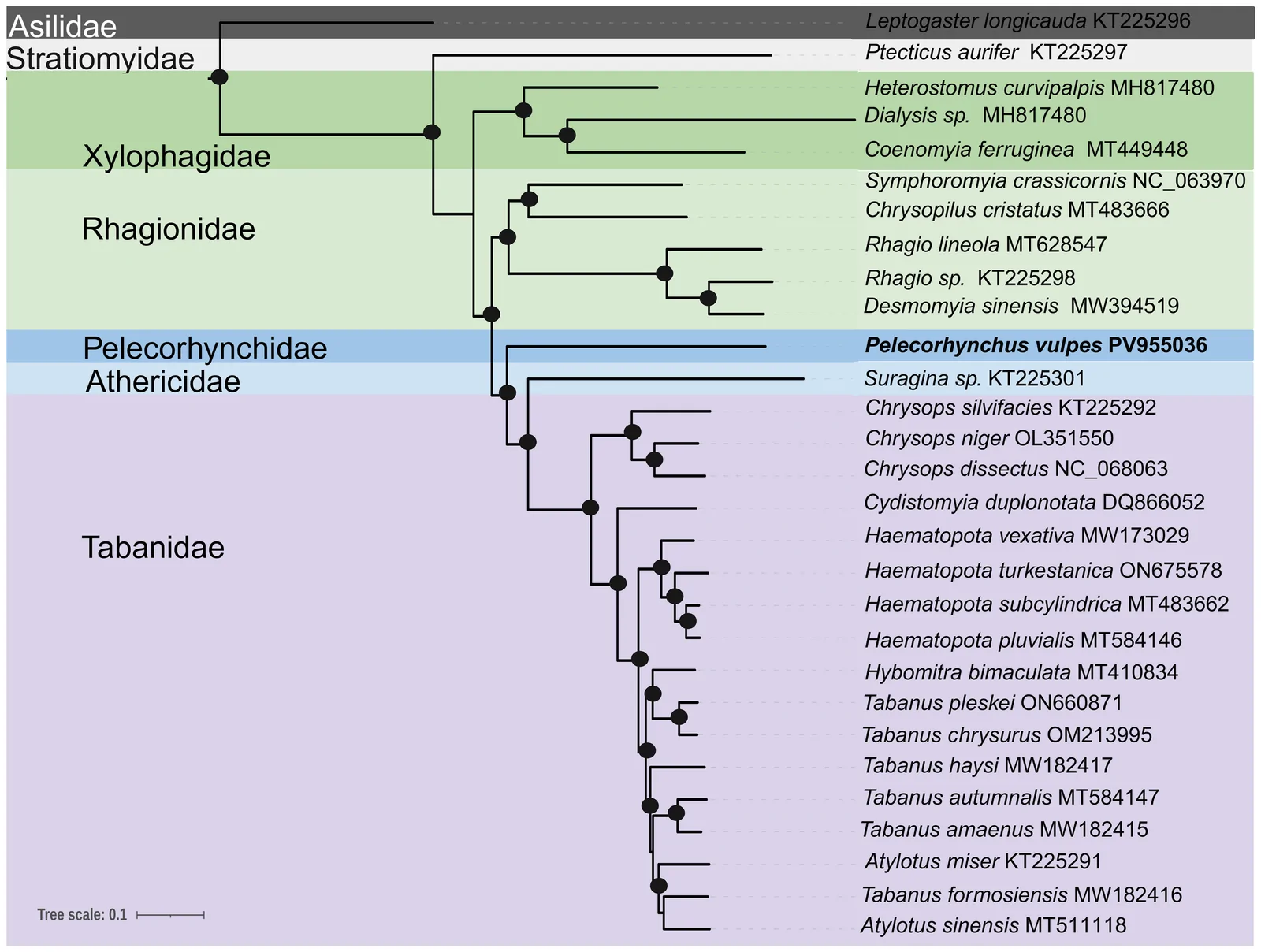
Taxonomic position of *Pelecorhynchus vulpes* within the phylogeny of Brachycera families inferred using Bayesian Inference (BI). Based on concatenated nucleotide sequences of 13 protein-coding genes (PCGs) and the ribosomal genes * rrnL* and *rrnS* (13,094 bp). *Leptogaster longicauda* (Asilidae) and *Ptecticus aurifer* (Stratiomyidae) were used as outgroups. Nodes with posterior probability (PP) ≥ 0.95 are indicated by filled circles. The scale bar represents the number of substitutions per site.

Phylogenetic relationships were inferred using both Bayesian inference (BI) and maximum likelihood (ML) methods based on a concatenated alignment of 13 protein-coding genes and two rRNA genes from 29 mitogenomes. Although only the Bayesian tree is shown (Fig. 5), both methods yielded congruent topologies. *Pelecorhynchus vulpes* (Pelecorhynchidae) was recovered as a sister to *Suragina* sp. (Athericidae) and formed a well-supported clade with Tabanidae (*Atylotus miser*, *A. sinensis, Chrysops dissectus, C. niger, C. silvifascies*, *Cydistomyia duplonotata*, *Haematopota subcylindrical*, *H. pluvialis*, *H. tukestanica*, *H. vexatina*, *Hybomitra bimaculate*, *Tabanus amaenus, T. autumnalis, T. chrysurus, T. formosiensis, T. haysi*), suggesting a closer phylogenetic relationship between Pelecorhynchidae and Tabanidae than with Xylophagidae (*Coenomyia ferruginea*, *Dialysis* sp., *Heterostomus curvipalpis*), which grouped separately. Nodes with posterior probability (PP) ≥ 0.95 are indicated with filled circles. The tree was rooted using *Leptogaster longicauda* (Asilidae) and *Ptecticus aurifer* (Stratiomyidae) as outgroups, forming a basal clade outside the Tabanomorpha, consistent with their established phylogenetic positions (Wang et al., 2016).

This study provides the first complete mitogenome of *P. vulpes* and represents the first mitogenomic resource for Pelecorhynchidae. The overall genome architecture, including gene content, order, and orientation, is highly conserved and conforms to the ancestral insect mtDNA pattern, as described for most Diptera (Cameron, 2014). This structural conservation suggests that mitogenome organization has remained evolutionarily stable across basal Brachycera, including the Tabanomorpha.

Comparative analyses indicated that the mitogenome of *P. vulpes* is highly similar to those of other tabanomorph families, particularly Tabanidae and Athericidae, in terms of genome size, gene arrangement, codon usage, and nucleotide composition. The high A+T content (78.4%) observed in *P. vulpes* falls within the typical range reported for dipteran mitogenomes and reflects the conserved mutational pressure across the infraorder (Cameron, 2014; Song, Yi & Yin, 2022). Likewise, the presence of conserved gene overlaps, such as those between *atp8/atp6* and *nad4/nad4L*, further supports the strong structural constraints acting on insect mitogenomes (Boore, 1999; Wang et al., 2016).

Despite this overall conservation, subtle differences in nucleotide skew and intergenic spacer length were detected, indicating lineage-specific evolutionary dynamics of the mitogenome. Variations in compositional bias across protein-coding genes, particularly within NADH dehydrogenase genes (ND) were observed in the *P. vulpes* mitogenome. Similar patterns have been reported in comparative mitogenomic studies of Diptera, in which ND genes often exhibited higher evolutionary rates and contributed disproportionately to phylogenetic signals (Zhang et al., 2015; Li & Li, 2022). In this context, the patterns observed here may contribute to phylogenetic signal within Tabanomorpha. However, as this study does not include explicit analyses of evolutionary rates or formal test of selection, evaluating potential associations with functional constrains would require additional analysis and is therefore beyond the scope of the present work.

Phylogenetic analyses support the sister group relationship between Pelecorhynchidae, Athericidae, and Tabanidae, consistent with previous studies based on morphological and multilocus molecular data (Wiegmann et al., 2003; Kerr, 2010; Morita et al., 2016). The placement of *P. vulpes* as a sister to Athericidae and closely related to Tabanidae reinforces the hypothesis that these families form a well-supported clade within Tabanomorpha (Morita et al., 2016). This result also corroborates earlier proposals that recognized Pelecorhynchidae as a distinct family rather than a subfamily within Tabanidae (Mackerras & Fuller, 1942) and highlights its evolutionary independence within the infraorder.

From an evolutionary perspective, the position of Pelecorhynchidae within this clade suggests that it may represent an early diverging lineage within the Tabanid assemblage. The combination of a conserved mitogenome structure and distinct phylogenetic placement indicates that major genomic rearrangements did not play a significant role in the early diversification of this group. Instead, diversification within Tabanomorpha may have been driven primarily by ecological and morphological innovations, with the mitogenome evolving under a relatively strong purifying selection. This pattern contrasts with that observed in other insect groups, in which extensive mitochondrial gene rearrangements are associated with rapid diversification (Boore, 1999).

However, it is important to note that although mitogenomic datasets provide valuable phylogenetic signals, they have inherent limitations. Mitogenomes are subject to compositional bias, heterogeneity in substitution rates, and maternal inheritance, which may affect phylogenetic inference, particularly at deeper evolutionary levels (Cameron, 2014; Song, Yi & Yin, 2022). In some dipteran lineages, mitogenomic analyses have produced inflated support values or topological inconsistencies compared to nuclear datasets (Li & Li, 2022). Therefore, while the results presented herein are consistent with previous studies, future research integrating nuclear genomic data is essential to further refine the phylogeny of the Tabanomorpha.

The biogeographic implications of our findings show that the phylogenetic relationship between Chilean *Pelecorhynchus* species and their Australasian counterparts supports a Gondwanan origin for this lineage, followed by vicariant divergence associated with the fragmentation of the southern continents. This pattern is consistent with the distribution of other basal brachyceran groups and reinforces the importance of southern hemisphere taxa in reconstructing the early evolutionary history of Diptera (Yeates & Wiegmann, 1999). Expanding taxon sampling, particularly among Australasian species, will be crucial for testing this hypothesis and better understanding the historical biogeography of Pelecorhynchidae.

In summary, this study provides the first mitogenomic characterization of Pelecorhynchidae and demonstrates the value of mitogenomes in resolving long-standing phylogenetic questions within the Tabanomorpha. By integrating new genomic data into a comparative framework, we provide novel insights into the evolutionary relationships, genomic evolution, and biogeographic history of this lineage, which is poorly understood. Overall, this study highlights the importance of incorporating underrepresented lineages, such as Pelecorhynchidae, to improve our understanding of evolution of early dipteran.

##  Supplemental Information

10.7717/peerj.21470/supp-1 Supplemental Information 1 Taxa included in this study with species names, GenBank accession numbers, genome sizes, references, and notes The taxa included in the study, organized by suborder, infraorder, superfamily, family, and subfamily. For each species, the corresponding GenBank accession number, mitochondrial genome size (bp), reference, and additional notes (e.g., complete or partial genome) are provided.

## Additional Information and Declarations

### Data Availability

The following information was supplied regarding data availability: The data is available in the Supplemental File. The raw sequence data are available at Genbank: PV955036, PRJNA1274070
